# Ecologically coherent population structure of uncultivated bacterioplankton

**DOI:** 10.1038/s41396-021-00985-z

**Published:** 2021-05-05

**Authors:** Conny Sjöqvist, Luis Fernando Delgado, Johannes Alneberg, Anders F. Andersson

**Affiliations:** 1 KTH Royal Institute of Technology, Science for Life Laboratory, Department of Gene Technology, School of Engineering Sciences in Chemistry, Biotechnology and Health, Stockholm, Sweden; 2 Åbo Akademi University, Faculty of Science and Engineering, Environmental and Marine Biology, Åbo, Finland

**Keywords:** Population genetics, Water microbiology

## Abstract

Bacterioplankton are main drivers of biogeochemical cycles and important components of aquatic food webs. While sequencing-based studies have revealed how bacterioplankton communities are structured in time and space, relatively little is known about intraspecies diversity patterns and their ecological relevance. Here, we use the newly developed software POGENOM (POpulation GENomics from Metagenomes) to investigate genomic diversity and differentiation in metagenome-assembled genomes from the Baltic Sea, and investigate their genomic variation using metagenome data spanning a 1700 km transect and covering seasonal variation at one station. The majority of the investigated species, representing several major bacterioplankton clades, displayed population structures correlating significantly with environmental factors such as salinity and temperature. Population differentiation was more pronounced over spatial than temporal scales. We discovered genes that have undergone adaptation to different salinity regimes, potentially responsible for the populations’ existence along with the salinity range. This in turn implies the broad existence of ecotypes that may remain undetected by rRNA gene sequencing. Our findings emphasize the importance of physiological barriers, and highlight the role of adaptive divergence as a structuring mechanism of bacterioplankton species.

## Introduction

Each liter of seawater contains around a billion bacterial and archaeal cells (bacterioplankton) that play central roles in biogeochemical cycles, marine food webs and ecosystem services [1, 2]. The diversity of aquatic prokaryotes is immense [3]. How this diversity is generated and structured is far from fully understood. Aquatic taxa are differentially distributed among habitats [4] and ribosomal RNA (rRNA) gene sequencing has shown that bacterioplankton communities are structured both in time and space [5–8]. While these studies have demonstrated that 16S rRNA gene clusters (OTUs), or even specific 16S rRNA gene sequences (amplicon sequence variants; ASVs), represent lineages adapted to different habitats, the 16S rRNA gene does generally not provide sufficient genetic resolution to reveal within-species (intraspecific) diversity patterns, since prokaryotes with identical 16S rRNA gene sequences may have highly divergent genomes and phenotypes [9].

Comparative genomics of isolates, as well as metagenomics on natural samples, have revealed sequence clusters of >95% average nucleotide identity (which has emerged as an operational delineation of prokaryotic species [10]); however, it is not known to what extent this intraspecific genomic variation represents neutral diversity vs. adaptation to different niches. Due to the technical challenges, relatively little is known about intraspecific structuring of microbes, not least in the marine environment. However, pioneering studies have shown that genetic content of single bacterial species may correlate with geographic distance [11], and that coexisting but ecologically differentiated strains may arise through resource partitioning [12]. For example, the picocyanobacterium *Prochlorococcus* has been demonstrated to display fine-scale sequence clusters associated with different regimes in temperature and light-intensity, and single-cell sequencing has revealed distinct genomic backbones within clusters and variation in accessory genes mainly between clusters [13]. The ubiquitous and most abundant type of organism in the ocean, the SAR11 clade, has undergone adaptive radiation in response to temperature [14]. Apart from “genome streamlining” [14, 15], it has been postulated that the ecological success of this organism is facilitated by its adaptive divergence into ecotypes specialized for specific environmental conditions [6, 16]. However, it is not known if intraspecific niche-differentiation is a general phenomenon in bacterioplankton or a characteristic of exceptionally abundant species. A deeper understanding of intraspecific diversity, sometimes referred to as “microdiversity” [17], is of crucial importance if we want to understand the ecology, evolution, and speciation of bacterioplankton, and of prokaryotes in general. Studying genomic variation within a species can also reveal genes involved in adaptation to specific environmental factors, providing new clues on gene functions and cellular mechanisms of adaptation.

The study of metagenomes has been predicted to offer a more realistic view of prokaryotic diversity [18, 19] as compared to PCR-based surveys of the 16S rRNA gene. Metagenomics offers different routes for addressing intraspecific variation. The first is to reconstruct genetic information of individual strains. By mapping reads from one or several samples to the reference genome(s) of a species, the gene complement and/or nucleotide sequences at variant positions of the constituent strains can be inferred [20–22]. This approach is promising, but challenging, especially in cases of many coexisting strains. The second approach does not aim at reconstructing strains, but rather uses the reads mapped to a reference genome to quantify intra- and intersample genomic variation of the species. This approach is more straightforward for analyzing population structure and works well also in case of highly complex pan-genomes. Schloissnig et al. [23] conducted pioneering cross-continental comparative analyses of human gut microbiomes using this approach and showed that there is more intraspecific genetic differentiation between habitats (human individuals) than within the same habitat over time. Similar approaches have been used by Nayfach et al. [11] and Delmont et al. [24] to show that gene content and amino acid composition, respectively, differ between oceanic regions within individual bacterial species.

Here we present the software POGENOM (POpulation GENOmics from Metagenomes) that, similarly to MIDAS [11], metaSNV [25] and inStrain [26] quantifies intraspecific genomic variation from metagenomic data. POGENOM differs from these softwares in that it takes as input a Variant Call Format (VCF) file, the standard file format for storing gene sequence variations. This allows the user to apply a variant caller of choice, rather than relying on an inbuilt algorithm. While MIDAS and inStrain provide genome-wide and gene-specific diversity estimates, metaSNV reports genome-wide population differentiation (Manhattan distance). POGENOM outputs both diversity and differentiation parameters, at the whole genome (nucleotide diversity and fixation index (*F*_ST_)) and at the gene level (nucleotide and amino acid diversity, pN/pS, and *F*_ST_ based on nucleotides and amino acids). POGENOM also provides permuted gene-wise *F*_ST_ values, facilitating significance tests on gene-wise differentiation.

We here use POGENOM to investigate patterns of genomic variation among a set of bacterioplankton species in the Baltic Sea. The Baltic Sea is a geologically young ecosystem with pronounced gradients of salinity, temperature, and nutrient concentrations, and is often used as a model for postglacial colonization and ecological differentiation [27, 28]. Marine macroorganisms display reduced species richness and intraspecific diversity towards the northern Baltic Sea, as the lower salinity of these waters impose more challenging conditions. Likewise, freshwater species diversity decreases with increasing salinity levels towards the south-west [29]. Moreover, population genetic studies have shown that species of fish and macroalgae have distinct genetic populations in the Baltic Proper (central Baltic Sea) compared to the Atlantic west of Sweden [30–32]. With respect to microorganisms, population genetic data across the salinity regimes is only available for one eukaryote: the marine diatom *Skeletonema marinoi* [33]. It is evident that the species is locally adapted and genetically differentiated into separate populations on each side of the Danish Straits, correlating with different salinity regimes and oceanographic connectivity. For bacterioplankton, community composition has been shown to vary significantly along the horizontal salinity gradient, as well as vertically along oxygen gradients, with the Baltic Proper being composed of a mixture of typical freshwater and marine taxa [34, 35]. Using metagenomic binning and fragment recruitment analysis, Hugerth et al. [36] showed that the prokaryotic organisms in the Baltic Proper are genetically differentiated from closely related marine and freshwater lineages while displaying high similarity to sequences from North American brackish waters, suggesting that the Baltic Sea prokaryotes are members of a global meta-community adapted to brackish conditions. However, it remains to be investigated whether the bacterioplankton species display genetically structured populations within the Baltic Sea ecosystem.

## Results

**Fig. 1:**
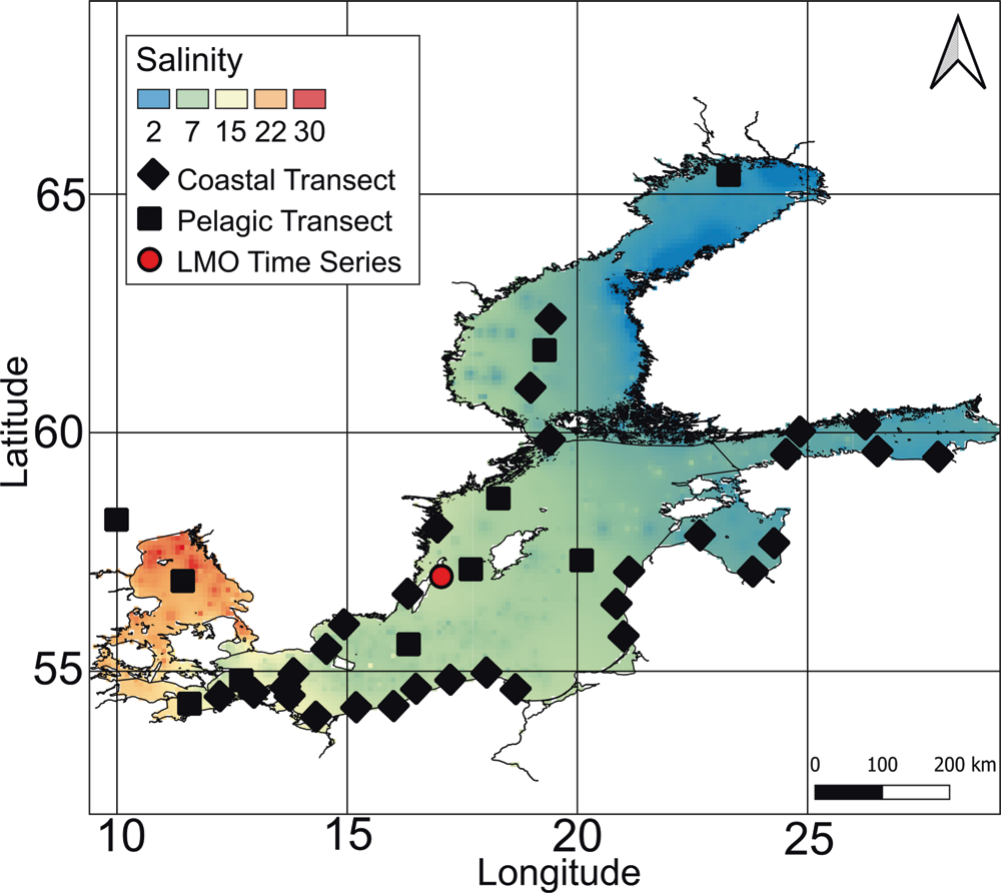
The Baltic Sea salinity gradient. Based on daily average surface salinity data from 2007 to 2017. Data retrieved from the Baltic Marine Environment Protection Commission (http://metadata.helcom.fi/). Sampling sites for the metagenomic data sets used in this study are indicated with symbols.

To improve understanding in intraspecific variation and revealing patterns in population genomic structure in bacterioplankton, we applied our newly developed software POGENOM on a set of MAGs that was recently reconstructed from a dataset of 123 water samples covering environmental gradients of the Baltic Sea [37] (Supplementary Table 1). This set includes 1961 MAGs that were clustered at 96.5% average nucleotide identity (ANI) into 352 species-level clusters [35]. These clusters are hereafter referred to as BACLs (BAltic Sea CLusters), and sometimes, for convenience, the term species is used, although these are not strict species. We selected a subset of 22 BACLs for our analyses, displaying sufficiently high coverage in at least ten surface water samples (see Methods). For each BACL, one representative MAG was used (with average estimated completeness and contamination of 91% and 3%, respectively; Table 1 and Supplementary Table 2). Metagenome reads from surface water samples from two transect cruises (Pelagic Transect 2014 (*n* = 10) and Coastal Transect 2015 (*n* = 34)) and from a 2-year time-series from one off-shore station (the Linnaeus Microbial Observatory (*n* = 22)) (Fig. 1 and Supplementary Table 1) were mapped to the MAGs [37, 38]. To lower the risk of including reads derived from other species, we only included reads mapping with >95% identity to the MAGs. We further used a median coverage depth threshold of ≥20X and a minimum coverage breadth of 40% to include a sample for a BACL. To avoid biases stemming from differences in coverage depth between samples, mapped reads were downsampled to the same median coverage (20X) for all samples.

**Table 1:** BACLs for which a representative MAG was included in the population genomic analysis and their overall SNV frequency (number of variant loci/genome size) and mean within-sample nucleotide diversity (π). The table is ordered by taxonomy.

Baltic Cluster	Genome size (bp)	Number variant loci	SNP frequency	Mean intra-sample π	Phylum	Family	Genus
BACL13	1404359	2954	0.0021	0.0006	Crenarchaeota	Nitrosopumilaceae	Nitrosopumilus
BACL27	1786241	26173	0.0147	0.0033	Actinobacteriota	Ilumatobacteraceae	BACL27
BACL17	1756084	22436	0.0128	0.0026	Actinobacteriota	Ilumatobacteraceae	UBA3006
BACL6	2791422	12221	0.0044	0.0011	Actinobacteriota	Ilumatobacteraceae	UBA3006
BACL112	1305743	13388	0.0103	0.0029	Actinobacteriota	AcAMD-5	ATZT02
BACL2	1227951	23983	0.0195	0.004	Actinobacteriota	Nanopelagicaceae	MAG-120802
BACL15	1276454	8011	0.0063	0.0015	Actinobacteriota	Nanopelagicaceae	Planktophila
BACL18	1767133	14674	0.0083	0.0016	Bacteroidota	Cryomorphaceae	TMED14
BACL8	2159345	6697	0.0031	0.0008	Bacteroidota	Flavobacteriaceae	MAG-120531
BACL327	1850878	26602	0.0144	0.0034	Cyanobacteria	Cyanobiaceae	PCC7001
BACL325	838244	15671	0.0187	0.0038	Proteobacteria	Pelagibacteraceae	NA
BACL144	1246612	17204	0.0138	0.003	Proteobacteria	Pelagibacteraceae	NA
BACL149	1310694	13757	0.0105	0.0026	Proteobacteria	Pelagibacteraceae	IMCC9063
BACL53	1312850	23119	0.0176	0.0032	Proteobacteria	Pelagibacteraceae	IMCC9063
BACL5	1242356	8386	0.0068	0.0016	Proteobacteria	Pelagibacteraceae	Pelagibacter
BACL262	1130444	12534	0.0111	0.0026	Proteobacteria	Pelagibacteraceae	Pelagibacter
BACL214	2228508	25432	0.0114	0.0024	Proteobacteria	Burkholderiaceae	UBA2463
BACL14	1388129	7055	0.0051	0.0013	Proteobacteria	Methylophilaceae	BACL14
BACL3	2958067	16697	0.0056	0.0013	Proteobacteria	Pseudohongiellaceae	OM182
BACL1	1513717	15731	0.0104	0.0018	Proteobacteria	D2472	D2472
BACL168	2226010	4876	0.0022	0.0005	Verrucomicrobiota	NA	NA
BACL9	1774586	12495	0.007	0.0018	Verrucomicrobiota	UBA3015	UBA3015

### Nucleotide diversity

**Fig. 2:**
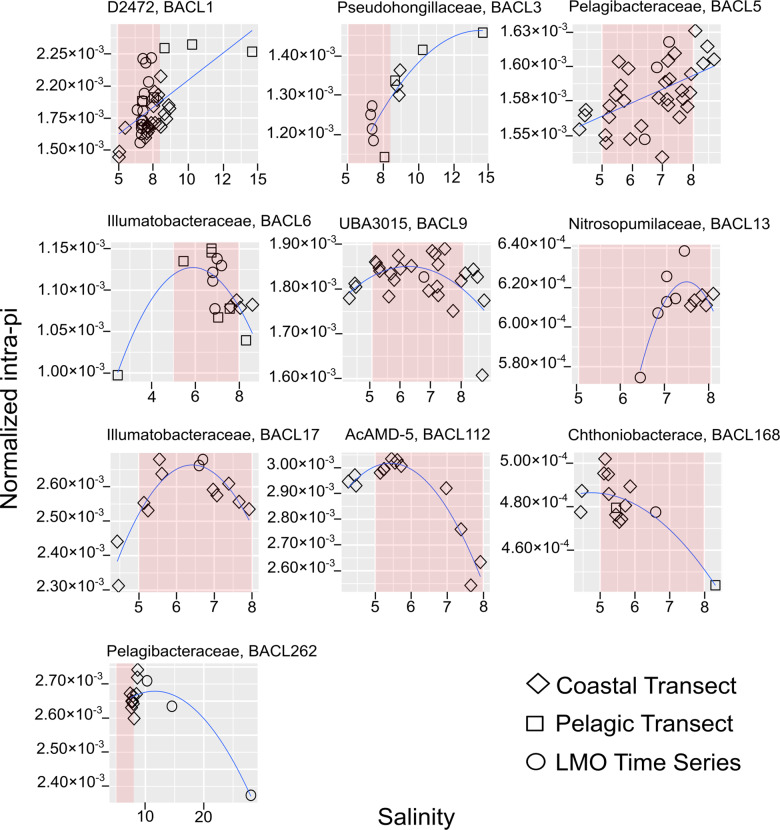
Intrapopulation π vs. salinity across the Baltic Sea ecosystem. Shaded area in red indicates salinity 5–8 (the horohalinicum [88]), to simplify the comparison of the graphs. Note different scales on *y*- and *x*-axes. All plotted genomes (10 of the 22 genomes) show statistically significant (*p* < 0.05) correlation with salinity, either using a linear (BACL1, BACL5) or quadratic regression model (all other BACLs), chosen based on the lowest AIC (Akaike information criterion) value. Complementary information (Supplementary Table 3) include statistical parameters for all models (also non-significant).

In total, 355,951 single-nucleotide polymorphisms were identified in the 22 genomes, with frequencies ranging from 0.0021 kbp^−1^ (BACL13) to 0.0195 kb^−1^ (BACL2). Mean within-sample nucleotide diversity (π), corresponding to the likelihood that two metagenome reads that overlap a position in the genome will differ at the position, ranging from 0.0005 (BACL168) to 0.0040 (BACL2) (Table 1). Since intraspecific genetic diversity tends to decline towards the extremes of a species’ niche-gradients [39], we investigated if any patterns in π across the salinity gradient could be detected. Indeed, eight out of the 22 genomes displayed a significant non-linear pattern (quadratic regression model) of within-sample π, while the π of two genomes (BACL1 and BACL5) best fit a linear regression model with salinity (Fig. 2, Supplementary Table 3). The observed non-uniform patterns of π are likely not caused by biases between the three sample sets, as no systematic grouping according to sample-set was observed (Fig. 2).

No strong seasonal trends in π were observed in the LMO time series data. However, a significant positive correlation was observed between the difference in seasonal time and difference in π for two genomes (BACL1 and BACL149) out of the five tested (≥8 samples required; Supplementary Fig. 1).

### Population genomic structure

**Fig. 3:**
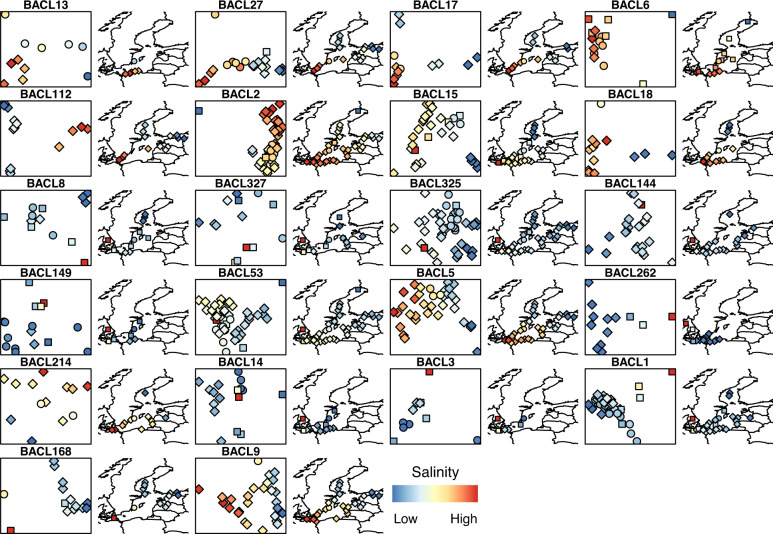
Population structure of Baltic Sea bacterioplankton. Left hand panel for each BACL represents a PCoA based on *F*_ST_ values, each data point is one sample. Right hand panel depicts the geographic location for each sampling point. The symbol colors indicate salinity values, with independent scales for the different BACLs. Circles = LMO Time series, squares = Pelagic Transect, diamonds = Coastal Transect.

**Fig. 4:**
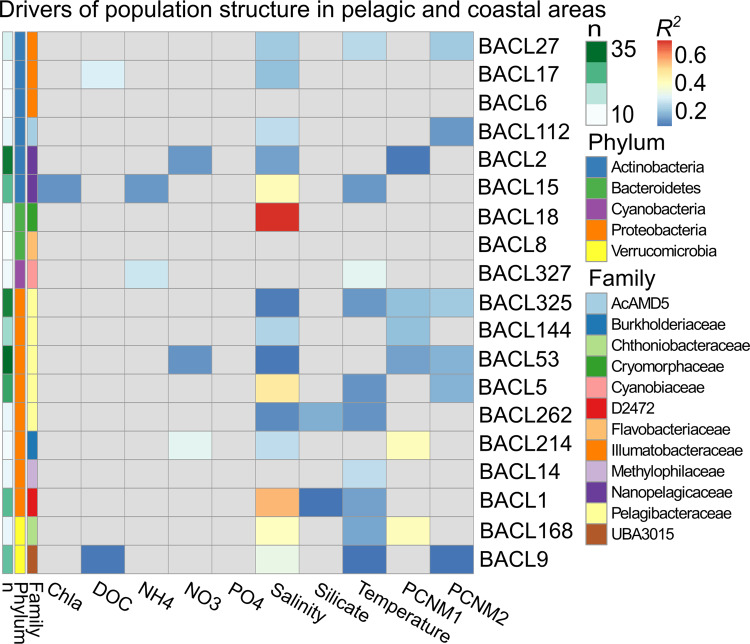
Environmental drivers of population structure in 19 prokaryotic BACLs across the pelagic and coastal transects. The number of samples (*n*) included for each BACL are indicated by the left-most column. Results stem from conditioned redundancy analyses. The PCNM variables represent the spatial relationship between sampling sites, reflecting pairwise geographic distances (shortest waterway distance). *R*^*2*^ values are colored from blue to red; only significant correlations are shown (*p* < 0.05).

Ordination of the samples based on pairwise fixation index (*F*_ST_)––a measure of population differentiation across samples––revealed a non-random population structure across the Baltic Sea for the majority of the analyzed genomes, with salinity correlating with the first principal coordinate in most cases (Fig. 3). When conducting a partitioned distance-based redundancy analysis (dbRDA) on the transect samples, salinity emerged as the most important driver of population structure: Fifteen out of the 19 genomes present in at least ten transect samples displayed a significant correlation between *F*_ST_ and salinity level (*p* < 0.05; Fig. 4), eight of which displayed the highest correlation to salinity. Temperature was the second most common driver of population structure, followed by DOC, NH4, and NO3. In four genomes, geographic distance (one of the Principal coordinates of neighbor matrices parameters PCNM1 and PCNM2) showed the highest correlation with population structure, indicative of isolation by distance. It should also be noted that the two genomes lacking significant environmentally correlated spatial population structure were among those with fewest included samples; with more samples such structure may have been detected also for these. Similarly, it is possible that a higher sequencing depth would reveal even clearer population structures, due to reduced noise in the *F*_ST_ computations. However, benchmarking on one genome showed that *F*_ST_ values calculated using the applied coverage correlated well (Pearson *R* = 0.88) with values obtained using four times higher coverage (Supplementary Fig. 2).

Of the four genomes that were present in at least eight of the LMO time-series samples, two (BACL1 and BACL149) displayed a significant correlation between *F*_ST_ and temperature (*p* < 0.001; data not shown). We did not observe any significant correlations between population structure and season, when accounting for the correlation with temperature.

**Fig. 5:**
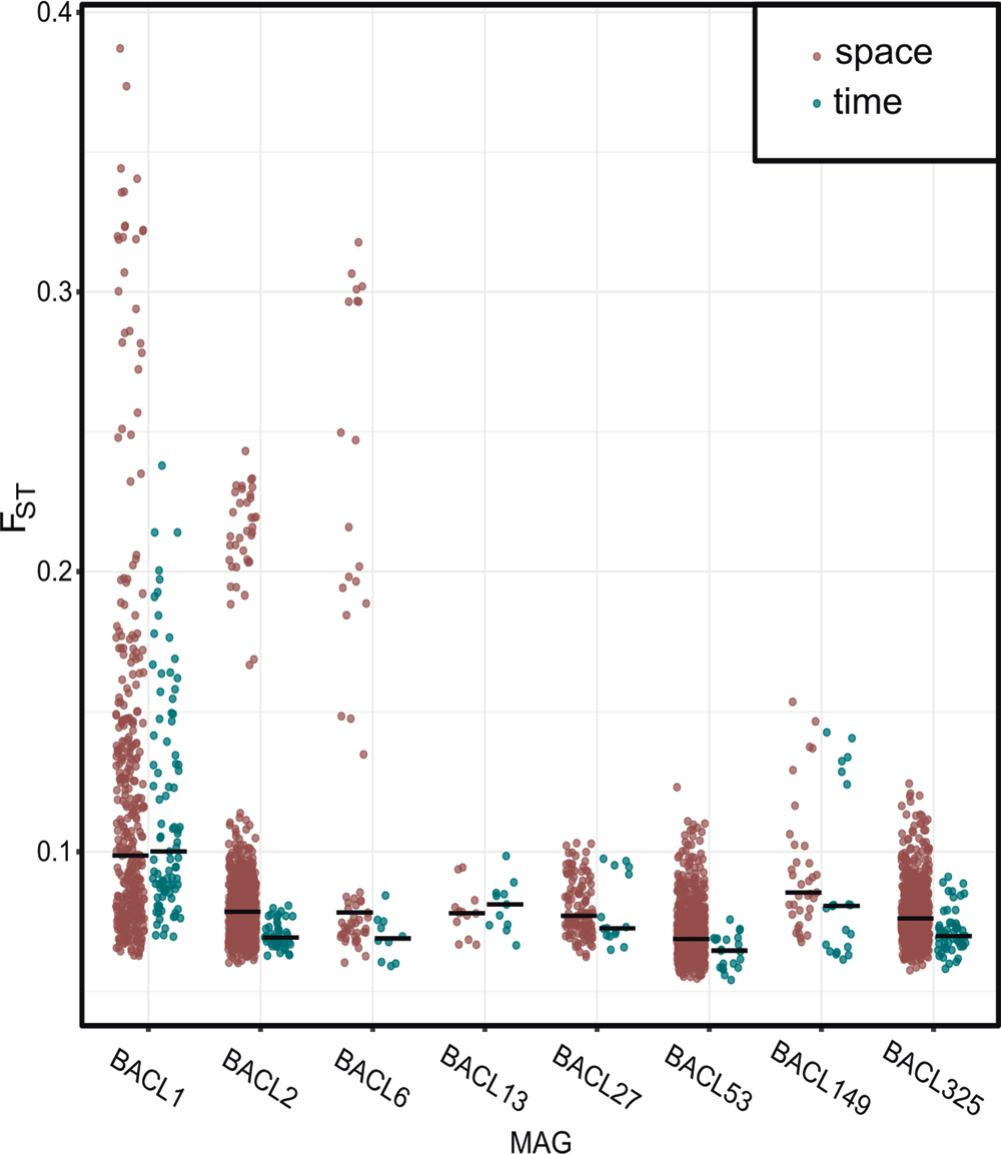
Comparison of *F*_ST_ values spatially across the Baltic Sea vs. over time at station LMO. Median values denoted with black lines. *p* values from Wilcoxon rank-sum tests comparing the distributions of *F*_ST_ values over time and space indicated by asterisks (<0.01**, <0.001***).

Among the BACLs for which a comparison between genomic differentiation over time and space was feasible, the magnitude of differentiation was greater spatially across the Baltic Sea than temporally at station LMO in six out of eight cases (Fig. 5). For four of them, the difference was statistically significant.

### Genetic variation at the gene level

**Fig. 6:**
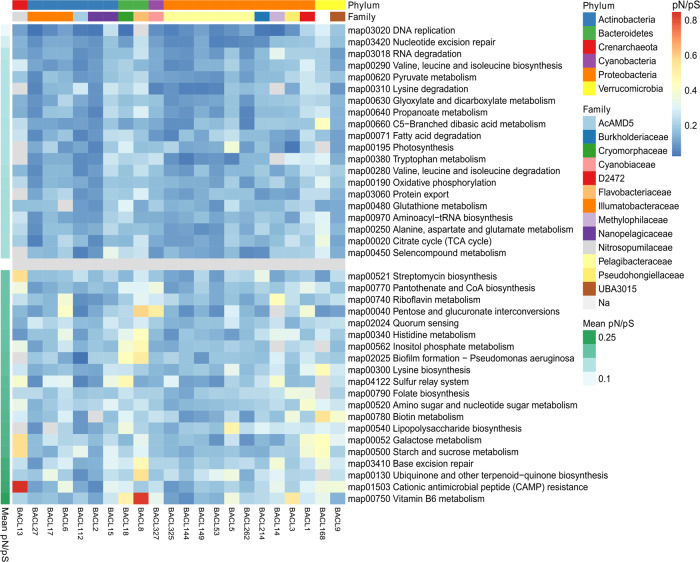
Summary of pN/pS values for KEGG pathways in 22 bacterioplankton genomes. Pathways sorted by mean pN/pS across genomes. The upper and lower panel consist of the twenty KEGG pathways with the lowest and highest mean pN/pS values, respectively.

The previous analyses reveal patterns of genomic variation at the whole genome level. To investigate patterns of selective constraints in these genomes, POGENOM estimates the ratio of non-synonymous to synonymous polymorphism rates (pN/pS) for each gene and sample, where low values indicate negative (purifying) selection and high values relaxed negative selection or diversifying selection. As expected, house-keeping genes (using a set of 36 single-copy core genes (SCGs) that are found in single-copy in nearly all known bacterial genomes [40]) had on average lower pN/pS values than other genes in most (20/22) genomes (Supplementary Fig. 2), reflecting stronger purifying selection in genes with core functions than in average genes [41, 42]. Likewise, comparing pN/pS values between genes belonging to different Kyoto Encyclopedia of genes and genomes (KEGG) [43] pathways showed that the pathways with lowest average pN/pS values (indicative of negative selection) tended to be associated with house-keeping processes such as transcription, RNA degradation, nucleotide excision repair and oxidative phosphorylation (Fig. 6, upper panel). Among the pathways with the highest pN/pS ratios (Fig. 6, lower panel), suggesting relaxed purifying selection or diversifying selection, we found several related to biofilm formation and antimicrobial synthesis and resistance. The pathway with the highest pN/pS value (0.52) in a single genome (BACL13) was cationic antimicrobial peptide (CAMP) resistance.

**Fig. 7:**
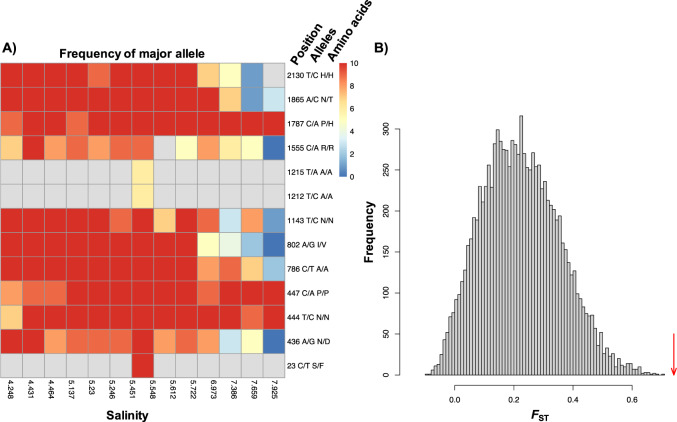
Example of genetic differentiation in a gene: ID = 302_3 of BACL262 (ABC-type proline glycine betaine transport system permease component). **A** Heatmap showing allele frequencies at different salinities along the transect, the color indicates the counts of the major allele in each sample (maximum = 10 since the counts were downsampled to this level, see Methods). Nucleotide position in the gene, major/minor alleles (nucleotides), and the resulting amino acid translations, are given to the right. **B** Distribution of permuted *F*_ST_ values for the gene based on 10,000 permutations. The red arrow indicates the actual *F*_ST_ value (0.74).

To identify environmental selection on specific genes, we focused on salinity, since this was the major driver of population structure in most BACLs. To facilitate the interpretation of population structure, POGENOM calculates *F*_ST_ at the individual gene level. For the 19 genomes that were present in at least ten transect samples, between 65 (BACL327) and 1245 (BACL18) genes displayed a positive correlation between *F*_ST_ and difference in salinity (Spearman correlation, FDR adjusted *p* < 0.05). These correlations may indicate that the genes themselves have undergone adaptation to the different salinity levels (or to environmental factors that co-vary with salinity) but could also reflect genetic hitchhiking, i.e., that an allele that differs in frequency between environmental conditions does so because allele(s) elsewhere on the genome have undergone selective sweeps. To increase the chance of identifying genes that truly have undergone positive selection in relation to salinity, we investigated gene-wise *F*_ST_ values for the pair of samples with the largest difference in salinity for each genome. We devised a permutation procedure, where a permuted *F*_ST_ value is calculated for each gene by shuffling variant loci over the genome while keeping the population differentiation constant at the genome-level (see Methods). This showed that a number of genes (1–32) in all but two BACLs displayed a higher *F*_ST_ (FDR adjusted *p* < 0.05) than expected by chance, given the genomes’ background levels of differentiation (Fig. 7 gives one example). The majority (91%) of these genes also displayed a positive correlation between *F*_ST_ and difference in salinity based on all transect samples. Pathway enrichment analysis indicated that the 151 genes that both displayed a significant *F*_ST_ for the salinity extremes and a correlation between *F*_ST_ and salinity difference were enriched in certain KEGG pathways: six pathways were enriched in at least two different BACLs each, and these were also enriched when considering all BACLs (*p* < 0.05; Table 2 and Supplementary Table 4). Four of these; Nitrogen metabolism (map00910), Alanine, aspartate and glutamate metabolism (map00250), ABC transporters (map02010) and Glyoxylate and dicarboxylate metabolism (map00630) were all enriched in two actinobacterial genomes each. The eggNOG category that was enriched in most genomes (*n* = 4) was Inorganic ion transport and metabolism (P).

**Table 2:** Summary of the KEGG pathway and eggNOG category enrichment analyses on the set of genes that displayed significant *F*_ST_ levels between the BACLs’ salinity extremes and significant correlations between *F*_ST_ and salinity difference.

BACL	Family	Number of significant genes	Significant KEGG pathways	Significant eggNOG categories
BACL27	Ilumatobacteraceae	2	map00910	P
BACL17	Ilumatobacteraceae	13	map00250,map02010,map00460	E,P
BACL6	Ilumatobacteraceae	26	map00020,map00630,map02024*,map00405,map02025	
BACL112	AcAMD-5	32	map03410,map00220,map00250,map00630,map00910,map00471	
BACL2	Nanopelagicaceae	18	map00984	P,
BACL15	Nanopelagicaceae	4	map02010	
BACL18	Cryomorphaceae	6	map03008	
BACL8	Flavobacteriaceae	2	map00550,map01502	M
BACL327	Cyanobiaceae	2	map00480,map00980,map00982,map00983	
BACL325	Pelagibacteraceae	1		
BACL144	Pelagibacteraceae	7	map00290,map00620,map00561,map00430	E
BACL53	Pelagibacteraceae	13	map00460,map00627,map00643,map00790,map04122	G
BACL5	Pelagibacteraceae	1	map00330	
BACL262	Pelagibacteraceae	16	map03060,map03070,map00680,map00190*	U,P*,C
BACL214	Burkholderiaceae	0		
BACL14	Methylophilaceae	0		
BACL1	D2472	3	map00220	
BACL168	NA	0		
BACL9	UBA3015	5	map00450	
All		151	map02010,map00910*,map03420,map00250,map00630*,map02024,map00460,map00220	P*,E

The analyses included the 19 BACLs present in at least ten samples across the transect. Shown pathways/categories had *p* values <0.05 in Fisher’s exact tests, and those highlighted with * had FDR adjusted *p* values < 0.05.

## Discussion

In this study we estimated genomic diversity and population differentiation for a set of uncultured aquatic prokaryotic species along environmental gradients across the Baltic Sea. We quantified population genomic indices, such as the intra-population diversity (π) and the fixation index (*F*_ST_), and quantified pN/pS ratios for genes belonging to different pathways across the study system to detect potential biases in selection pressures. With these analyses, we obtained information about environmental drivers of population structure and indications on functional traits under selection. Such an exercise is now significantly streamlined with the software POGENOM, calculating the above parameters automatically. With intra-population diversity (π), we refer to the average nucleotide diversity of a population, while the fixation index (*F*_ST_), measures the differences in allele frequencies between pairwise populations. *F*_ST_ was originally designed for diploid, sexually reproducing organisms [44] where a value close to 1 is interpreted as substantially restricted gene flow between populations. However, the concept of *F*_ST_ involves no obligate condition of sexual reproduction, as it simply compares allele frequencies between two populations and is thus as valid for asexually reproducing prokaryotes [45]. Constraints in gene flow as apprehended in sexually reproducing organisms may for prokaryotes be seen as constraints in homologous recombination and/or effects of environmental sorting of genetic material, leading to skewed allele frequencies between populations.

Our analyses showed that the majority of the BACLs had a genomic population structure significantly correlating with salinity across the Baltic Sea, i.e., that the diverse and environmentally structured bacterioplankton communities that have been described earlier in this ecosystem [34, 46–48] are even further differentiated at the species level. A few BACLs displayed statistically significant isolation by distance (IBD), similar to the findings in Nayfach et al. [11]. However, variation partitioning indicated that geographic distance was the strongest driver of population structure only in four cases out of 19 after taking environmental factors into account (Fig. 4). In cases where stable differential selection is sustained, as in the Baltic Sea, and where geographic distances are rather small, isolation by adaptation (IBA) can spur population structure within a species [49, 50]. When comparing the extent of differentiation across space and time, *F*_ST_ values were on average higher for geographically than temporally separated populations, which probably reflects that salinity is a stronger driver of population structure than the environmental factors that covary with season. Hence, our data indicate that intraspecific genomic differentiation is more pronounced over the spatial than the temporal scale in the Baltic Sea ecosystem, analogous to what has previously been proposed for species sorting at the community level [35]. However, a couple of BACLs displayed temporal population structures significantly covarying with temperature, exemplifying that population structure in marine bacterioplankton also adhere to variations in niches at the same geographic location. Combined, these results show that aquatic bacterial species typically diverge from the null hypothesis of panmixia and that populations are structured by species-specific environmental drivers. This in turn implies the broad existence of ecotypes [51] that may remain undetected by 16S rRNA gene sequencing.

Earlier comparative genomic studies have shown remarkable differences in gene content of bacterioplankton belonging to the same species and concluded that the flexible part of the genome is modified by horizontal gene transfer in response to selective forces [11, 52–55]. Our results suggest that environmental selection in bacterioplankton also selects for and preserves specific alleles in the existing genes. Delmont et al. [24] suggested an evolutionary mechanism for such conservation of genetic heterogeneity by emphasizing the role of adaptive selection, exemplified by the cosmopolitan SAR11 clade. The same authors showed a partitioning of SAR11 populations in concordance with large-scale oceanic temperatures, suggesting that environmental selection is of central importance even at the microdiversity level in marine bacterioplankton. Our study shows that environmentally driven population structure is not limited to species of certain clades, but rather appears to be a general pattern for bacterioplankton. We, also, observe this in several species of SAR11 (Pelagibacteraceae), but also in several other taxa. For example, BACL1, belonging to the cosmopolitan Gammaproteobacterial clade SAR86, displayed a population genomic structure mainly driven by salinity in the Baltic Sea ecosystem. Another abundant group in the Baltic Sea, the Actinobacteria, are also mostly driven by salinity according to our analyses. Actinobacteria are one of the most abundant types of freshwater bacteria globally and comprise multiple different clades and species-level clusters [56, 57]. Recent discoveries on their marked microdiversification may explain their success in environmentally variable conditions [58]. The Baltic Sea also hosts an abundant community of different cyanobacteria species. An example is the cosmopolitan genus *Synechococcus*, which is prevalent in the Baltic Sea during summer or when the temperature reaches >15 °C [59]. The environmental association analysis showed that the population structure of the *Synechococcus sp*. analyzed here (BACL327) was mainly driven by differences in sea surface temperature across the Baltic Sea. A marked difference between the situation in the Baltic Sea and in the ocean, however, is that in the ocean, temperature appears to be the main factor structuring bacterioplankton, both at the community [60, 61] and population [24] level, while in the Baltic Sea, salinity is the primary driver.

Empirical studies on marginal populations of macroorganisms living close to the species range limits show decreased genetic diversity, thus lowering their adaptive potential to rapidly changing environmental conditions [28, 62]. This has been observed in several marine macroorganisms adapted to the brackish conditions in the Baltic Sea, where the populations residing inside the Baltic display lower intra-species diversity than their Atlantic founder populations [28]. In contrast, several of the BACLs displayed a significant hump-shaped curve in intra-population diversity (π) along the salinity gradient. However, our previous study based on MAGs from station LMO indicated that the bacterioplankton of the Baltic Sea are members of a globally distributed brackish metacommunity, rather than locally adapted freshwater and marine taxa [36]. Thus, unlike most macroorganisms in this ecosystem, planktonic prokaryotes residing in the Baltic Sea were likely adapted to brackish conditions already when they entered the system, consistent with the hump-shaped curves in diversity. Whether most of the intraspecific variation and niche differentiation that we see within the different BACLs were gained after the populations immigrated, or was in place already before, as a set of strains with different genetic make-up and ecological niches, remains elusive.

Recently, proteome differences between some freshwater prokaryotes and their closest marine relatives were described, with a larger proportion of acidic and a lower number of alkalic amino acids in the proteome of the marine representative of each pair, compatible with a “salt-in” strategy earlier observed in halophilic prokaryotes [63]. This indicates that adaptations changing the chemical properties of the proteome may be important for crossing the freshwater - marine boundary [64]. However, none of the 22 BACLs displayed a significant correlation between salinity level of the sample and frequency of either acidic or alkaline amino acids in the population proteome, as deduced from the per-sample single amino acid variants (SAV) frequencies output by POGENOM (data not shown). Thus, adaptations altering the physicochemical properties of the proteome does not seem to be the major driver behind the genomic differentiation we observe within the Baltic Sea region for these populations. It may however have been important for facilitating the transition from freshwater or marine to brackish conditions in the first place.

The gene-wise pN/pS analysis addressed patterns of constraints in selection, not directly related to the environmental gradients. While we observed relatively low pN/pS ratios reflecting purifying selection for genes belonging to KEGG pathways related to housekeeping functions, indications of diversifying selection were observed in other parts of the functional spectrum. For instance, the cationic antimicrobial peptide (cAMP) resistance pathway exhibited the highest pN/pS value in a single BACL (BACL13). Antimicrobial peptides (AMPs) play important roles in host defense against microbial infections by weakening the membranes of the microbes, subsequently killing them. AMPs belong to a universal set of defense molecules synthesized across the domains of life [65] and are known to be produced by for example molluscs, crustaceans, ciliates, phytoplankton and bacteria in the marine environment [66–70]. Biosynthesis of streptomycin, a well-known antibiotic, which is coupled to inositol phosphate metabolism as part of the molecule is synthesized via myo-inositol. Both of these pathways displayed among the highest pN/pS ratios across BACLs. Interestingly Schloissnig et al. [23] showed elevated pN/pS ratios for antimicrobial resistance genes in the human gut microbiome. Our findings of elevated evolution in both synthesis and defense genes for antimicrobials suggest that adaptation related to chemical warfare is of central importance also for aquatic bacterioplankton, and raises an unexpected parallel to the human gut ecosystem.

The gene-wise *F*_ST_ analysis revealed a small number of genes in most BACLs displaying higher genetic differentiation between the salinity extremes than expected by chance, given the genomes’ background levels of genetic differentiation, i.e., the genes were enriched in loci displaying strong genetic differentiation between the samples with lowest and highest salinity. This indicates convergent evolution in multiple strains occupying one or both of the locations, either by the same mutations occurring independently in multiple strains, or by homologous recombination of a genomic segment between the strains, followed by selection. The fact that the polymorphisms of many of the loci displaying high differentiation in these genes were synonymous (i.e., did not cause a change in amino acid), argues for the homologous recombination scenario. Moreover, in several cases, multiple genes adjacent on the genome displayed significant *F*_ST_ values, congruent with the recombination scenario. This may in part be attributed to our approach for identifying genes under selection, which is likely to miss cases where the allele frequency of just a single (or very few) sites in a gene has been altered by selection. Among the pathways enriched in highly differentiated genes we find ABC transporters (map02010). This may reflect genetic adaptations to differences in concentrations in inorganic and organic nutrients across the Baltic Sea salinity gradient. One of the ABC transporters was glycine betaine/proline transport system permease protein (in BACL262; Fig. 7). Glycine betaine is a widely used compatible solute (osmoprotectant) in bacteria and is imported or synthesized in response to hyperosmotic stress [71]. A previous metagenomic study found genes for this transporter to be differentially abundant across the Baltic Sea [8]. Another enriched pathway was nitrogen metabolism (map00910). A closer look at this pathway showed that the significant genes are mainly related to glutamine and glutamate synthesis (Supplementary Fig. 4). Glutamine and glutamate both act as osmoprotectants in several bacteria including marine species [72–75] and the differing allele frequencies across the salinity regimes in the genes synthesizing them may reflect adaptations related to underlying enzyme kinetics.

## Conclusions

Facilitated by our recently developed program POGENOM, we show that populations of multiple bacterioplankton clades are genomically structured, even within the same ecosystem. Genomic differentiation within species correlated with environmental variables such as salinity, temperature and nutrient levels across spatial dimensions when accounted for geographic distance. This emphasizes the role of isolation by adaptation rather than isolation by distance as a driving force for speciation of aquatic prokaryotes. Population genomics analysis based on metagenomics data will undoubtedly lead to a deeper understanding of the ecology and evolution of important bacterioplankton species, which is of central importance when learning about how species adapt to new environmental conditions and what their adaptive potential is in the face of Global Change.

## Methods

### POGENOM software

POGENOM takes as minimal input a file of the variant call format (VCF). This is generated by mapping one or several metagenome samples against a reference genome with a read aligner such as Bowtie2 [76], BWA [77] or MOSAIK [78] and calling variants using a variant caller such as GATK [79] or Freebayes [80]. POGENOM calculates the nucleotide diversity (π) within each sample. If multiple samples have been mapped, the fixation index (*F*_ST_) is calculated for all pairs of samples. If, in addition to the VCF file, an annotation file of the General Feature Format (GFF) is provided, gene-wise π and *F*_ST_ will be calculated. If, further, the genome sequence is provided in the GFF file or in a separate FASTA file, amino acid frequencies will be calculated for each codon position in each gene and sample, and gene-wise π and *F*_ST_ will be calculated also at the amino acid level. Now also non-synonymous to synonymous polymorphism rates (pN/pS) will be calculated for each gene and sample. Optionally, permuted gene-wise *F*_ST_ values can be calculated. POGENOM has several optional parameters, such as minimum read depth for a locus to be included for a sample, minimum number of samples with minimum read depth for a locus to be included at all, subsampling to a given read depth, splitting of haplotypes into individual SNVs in case haplotype variant calling was applied, etc. A complete description on how the different parameters are calculated can be found in the Supplementary Information. POGENOM was implemented in Perl. Source code and documentation, and a pipeline for automatic generation of input data (Input_POGENOM), are available at https://github.com/EnvGen/POGENOM.

### MAG and shotgun sequencing data

In total 66 (10 pelagic, 34 coastal, and 22 time series) metagenomic samples, quality filtered as described before [37], were used for population genomic assessments of 22 MAGs. The MAGs were selected based on being prevalent in these samples based on data from Alneberg et al. [37]. Each MAG represents one unique species-level cluster (BAltic Sea CLuster; BACL) and no pair of MAGs in this set have >80% average nucleotide identity (ANI). MAGs were taxonomically annotated using GTDB-tk [81] v.0.3.2 using v.89 of the GTDB [82]. Gene calling was conducted with Prokka [83]. The shotgun data used for the population genomic assessments are derived from two cruises: Pelagic Transect 2014 [38] and Coastal Transect 2015 [37], as well as from two years of time-series data from the Linnaeus Microbial Observatory (LMO) station [37]. Sampling, DNA extraction and sequencing procedures have been described earlier [37, 38], but very briefly, surface water was filtered through 0.2 um filters, either directly (transect samples), or after pre-filtration through 3.0 um filter (time-series samples), DNA was extracted from filters and shotgun sequenced on a HiSeq (Illumina) with on average 48 million read-pairs per sample.

### Variant calling

The Input_POGENOM pipeline was used for automatic generation of input files for POGENOM, i.e., VCF files. Briefly (more information provided in the online documentation), Input_POGENOM is a Snakemake [84] pipeline that uses Bowtie2 [76] for read mapping to the reference genome and Freebayes [80] for variant calling. Bowtie2 v.2.3.4.3 was used and the ‘bowtie2_params’ of Input_POGENOM was set to “–ignore-quals –mp 1,1 –np 1 –rdg 0,1 –rfg 0,1 –score-min L,0,-0.05”, corresponding to a 95% identity threshold between read and genome. The parameters ‘min_coverage’ were set to 20 and ‘min_breadth’ to 40, i.e., only samples where the genome displayed ≥20X median coverage depth and ≥40% coverage breadth (fraction of genome covered by at least 1 read) were included for the genome. After mapping, the read-mapping (BAM) files are downsampled to the same coverage (to ‘min_coverage’) using samtools (v.1.9; [85]). Freebayes v.1.3.1 was used for the variant calling, which is run once per genome, after combining the BAM files from the approved samples into a multi-sample BAM file. The ‘freebayes_parameters’ was set to “-C 4 -p 1 –pooled-continuous –read-max-mismatch-fraction 0.05 –min-alternate-fraction 0.01 -q 15”, meaning that a SNV was called only when the variant allele was supported by ≥4 reads and with an allele frequency of ≥1%. Input_POGENOM was further run in ‘prefilt’ mode, meaning that it first estimates the coverage of a genome in a sample by only mapping a subset of the reads, in order to fastly eliminate samples unlikely to reach sufficient coverage.

### POGENOM runs

POGENOM v.0.8.2 was run with the parameter settings --min_count 10, --subsample 10, and --min_found 1 on the VCF file of all approved samples for each MAG. In other words, it included for a sample only those loci with allele counts ≥10 (i.e., with ≥10 overlapping reads), and for loci with counts >10, it downsampled to counts = 10. And overall, it included only those loci fulfilling the --min_count conditions for at least one sample. Although we used --min_found 1 here, it may be preferable to set it to the number of samples when for example comparing π between samples for the same genome. For comparing actual and permuted gene-wise *F*_ST_ values, POGENOM was run on only the pair of samples with extreme salinities for each MAG by specifying these samples using the --sample_file parameter, otherwise using the same parameter setting as above except also --fst_perm 10000, meaning that 10,000 permuted *F*_ST_ values were computed for each gene.

### Environmental association analysis

Nucleotide diversity was compared against salinity across the Baltic Sea using linear and quadratic regression models. The best model for each MAG was chosen based on AIC values for linear vs. non-linear models. Nucleotide diversity was also studied over time in the LMO data set using Spearman’s rank correlation. For the above analyses, and for the data presented in Table 1, we used normalized genome-wide π, calculated as described in the Supplementary Information. Relating to population structure we conducted environmental association analyses using a global dbRDA (distance-based redundancy analysis) followed by a conditioned analysis. This allowed us to disentangle the relative contribution of different independent variables in driving seascape genomic structure. The global dbRDA was conducted using all environmental variables (salinity, temperature, ammonia, nitrate, phosphate, silicate, chlorophyll a and dissolved organic carbon [and time for LMO samples]). *F*_ST_ matrices were subjected to an unconstrained Principal Coordinates Analysis (PCoA) and the PCoA-axes were used as dependent input in the dbRDA. Regression coefficients in the dbRDA are reported as adjusted values of multiple determination (*R*^2^-adj.). Statistical significance of the global dbRDA was evaluated using the permute-function from vegan and by performing an Anova (by “term”, 999 permutations) on the dbRDA result to assess the statistical significance of each variable. The conditioned analysis was only conducted in case the global dbRDA showed statistically significant explanatory power (*p* < 0.05) to avoid Type I error and overestimation of the explained variance [86]. Prior to the conditioned dbRDA we performed a forward selection procedure where variables are added to the model consecutively. The selection stops when adding a variable no longer improves the overall model (threshold, *p* < 0.05). Statistical significance of conditioned individual fractions (i.e., marginal effects) was evaluated by an Anova (by “margin”; 999 permutations). Time was transformed to PCNM-variables (Principal coordinates of neighbor matrices) by first conducting a PCoA on the matrix for time differences. All eigenvectors with positive values were included in the following RDA analysis where the *F*_ST_ matrices of respective MAGs were used as response data. All PCNM variables displaying significant correlation with population structure over time were included side by side with environmental variables in the global dbRDA. Likewise, geographic distance (shortest waterway distance) was transformed to PCNM-variables and used as explanatory variables in the dbRDA.

### KEGG pathway analysis

The genomes were functionally annotated using online eggNOG-mapper [87] which assigned the genes to KEGG pathways, KEGG modules, KEGG orthologs, eggNOGs, and eggNOG functional categories. For the gene-wise pN/pS and *F*_ST_ analyses, only KEGG pathways that we judged relevant for microbial genomes were included; the pathways belonging to categories 1.1–1.12, 2.1–2.4, 3.1, 4.4–4.5, and 6.11 (https://www.genome.jp/kegg/pathway.html#genetic). For the pN/pS analysis, only pathways that resulted in pN/pS values for >90% of the BACLs were included. For the KEGG pathway enrichment analyses, each pathway present in the BACL was checked for overrepresentation of significant genes using Fisher’s exact test. The *p* values from these tests were subsequently adjusted for multiple testing using False Discovery Rate. For genes assigned to multiple pathways, all of the assignments were used. The eggNOG functional category enrichment analyses were done in the same way. The KEGG pathway map of Supplementary Fig. 4 was generated using the KEGG database online resource (https://www.genome.jp/kegg/).

### Permutation analysis of gene-wise *F*_ST_ values

POGENOM calculates permuted gene-wise *F*_ST_ values for a pair of samples by shuffling all variant loci for the genome so that each gene will get a new set of loci (with their associated allele frequencies) while having the same number of variant loci as in the original data set. Subsequently, gene-wise *F*_ST_ values are calculated based on the shuffled data. This procedure is repeated many times and *p* values can be calculated by comparing the actual *F*_ST_ value with the distribution of permuted *F*_ST_ values for each gene; here we defined the *p* value as (1 + the number of permuted *F*_ST_ values ≥ the actual *F*_ST_ value)/(the number of permutations) and we performed 10,000 permutations.

## Supplementary information

Suppl. figures and info

Supl tables

## Data Availability

The MAG sequences as well as the preprocessed sequencing reads from the LMO Time Series 2013–2014 and Coastal Transect 2015 samples were published before (Alneberg, 2020) and are available at ENA hosted by EMBL-EBI under the study accession number PRJEB34883. The preprocessed sequencing reads from the Transect 2014 samples were also published elsewhere (Alneberg, 2018) and are available at ENA under the study accession number PRJEB22997. Source code and documentation for POGENOM are available at https://github.com/EnvGen/POGENOM.
