# Supplementary material for: Ecologically coherent population structure of uncultivated bacterioplankton
Source: ISME J. 2021 May 5;15(10):3034–49. doi: 10.1038/s41396-021-00985-z (PMC8443644; doi:10.1038/s41396-021-00985-z)

Supplementary Information

Table of contents

1. Calculation of population genetic parameters in POGENOM

    1.1. Nucleotide diversity, π

    1.2. Fixation index, F_{ST}

    1.3. Amino-acid level π and F_{ST}

    1.4. *pN/pS*

2. Supplementary figures

1. Calculation of population genetic parameters in POGENOM

1.1. Nucleotide diversity, π

Nucleotide diversity (π), sometimes called heterozygosity, is defined as the average number of nucleotide differences per site between any two sequence reads chosen randomly from the sample population ($0 \leq \pi < 1$). POGENOM calculates π of a single locus according to Schloissnig et al. [23]:

$$\pi_i = \sum_{B_1 \in \{ACTG\}} \sum_{B_2 \in \{ACTG\} \setminus B_1} \frac{x_{i,B_1 S_1}}{c_i} \frac{x_{i,B_2}}{c_i - 1}$$

where $x_{i,Bj}$ is the count of nucleotide B_j at position i in the genome for the sample, and c_i is the total coverage (sequence depth) at position i for the sample. To calculate a genome-wide π, π is averaged over all loci by summing all π_i and dividing by the genome size. Loci not included in the VCF file are assumed to lack diversity (to have $\pi_i = 0$; but see the normalised π below). To calculate a gene-wise π, π is instead averaged over all loci within the gene, including the start codon but excluding the stop codon. The π calculation also works for alleles >1 bp (in case a variant caller was used that output haplotypes), then instead basing the calculations on counts of haplotypes (oligomers) rather than nucleotides for loci where alleles >1 bp are reported in the VCF file. POGENOM can also split counts of haplotypes into counts of individual nucleotides, if this is preferred (this is the default behaviour). When the splitting is applied, POGENOM will remove loci of individual positions resulting from the splitting that do not contain any variants (this may be the case for internal haplotype positions), from the pool of variant loci. For gene-wise π, π will for a gene and a sample per default be set to N/A if one or several loci included in the VCF file for the gene have missing data for the sample, to avoid biases between samples for the gene due to missing data.

A locus not reported in the VCF file can be missing because no genetic variation was observed, but also because the locus did not have sufficient sequence depth coverage in the pool of samples when running the variant calling. The latter can lead to π values being biased downwards (since these loci are assumed to have $\pi_i = 0$) and can skew the comparison of π between genomes (but should however not affect comparisons between samples for the same genome, if multi-sample variant calling was conducted). The π for a sample may also be deflated if some loci included in the VCF have lower coverage for the sample than the threshold specified by the '--min_count' parameter, since these loci will not be included in the π calculation. Finally, loci included in the VCF file but not fulfilling the '--min_count' criterion in at least the number of samples specified by the '--min_found' parameter, will also be excluded from the calculation, further deflating the π. In order to adjust for these potential sources of errors, a normalised genome-wide π is also calculated for each sample by dividing the genome-wide π with an estimated completeness factor for the sample. The completeness factor is based on the assumption that loci with sufficient coverage (fulfilling the --min_count cutoff) in one sample are independent from those with sufficient coverage in another sample. Thus, the loci covered in sample 1 can be treated as a random subset of the genome and used to assess the completeness of sample 2, by calculating the fraction of loci covered by sample 1 that are also covered by sample 2. The completeness for a sample is assessed this way using all other samples, and the completeness factor is the average of these assessments. The normalised

genome-wide π is not used for F_{ST} calculations since missing variant loci should affect intra- and intersample π equally and have little influence on the F_{ST} (see calculations below).

1.2. Fixation index, F_{ST}

To calculate the fixation index (F_{ST}) for each pair of samples, the intersample π has to be calculated. For a single locus this is calculated according to[23]:

$$\pi_{i,S_1,S_2} = \sum_{B_1 \in \{ACTG\}} \sum_{B_2 \in \{ACTG\} \setminus B_1} \frac{x_{i,B_1,S_1}}{c_{i,S_1}} \frac{x_{i,B_2,S_2}}{c_{i,S_2}}$$

where $x_{i,Bj,Sk}$ is the number of nucleotide B_j observed at position i in the genome in sample S_k and $c_{i,Sk}$ is the coverage of position i in sample S_k. The inter-sample π is then calculated for the whole genome (or gene) by summing π_i for all loci (or loci inside the gene) and dividing by the genome (or gene) size.

F_{ST} is then calculated according to:

$$F_{ST} = 1 - \frac{mean\,(\pi_{intra\ sample})}{\pi_{inter\ sample}} = 1 - \frac{(\pi_{S_1} + \pi_{S_2})/2}{\pi_{S_1,S_2}}$$

where for genome-wide F_{ST} , the calculation is based on genome-wide intra- and intersample π values, while for gene-wise F_{ST} , it is based on gene-wise π values. For both types of F_{ST} calculations, only loci for which both samples in the pair have data will be considered for the intra- and intersample π calculations. If no such loci are present, or if the intersample π is zero (which only happens if also both of the intrasample π are zero), F_{ST} will be set to NA.

For permuted gene-wise F_{ST}, the variant loci are randomly redistributed among the genes in a way such that each gene will obtain a new set of variant loci, with their associated allele frequencies, but will have the same number of variant loci as in the original case. The randomisation is done this way for every pair of samples (loci will be redistributed the same way for both samples in the pair). As for the F_{ST} calculations above, only loci for which both samples in the pair have data will be included.

1.3. Amino-acid level π and F_{ST}

Gene-wise amino acid π is calculated based on the variant loci within genes, including the start codon but excluding the stop codon. Amino acid π for a single locus is calculated by modifying the gene sequence according to each detected allele (one at a time) for the locus in the sample and translating the modified gene into a peptide (based on the genetic code file). The counts of each unique peptide will then be used for the calculations of intra- and intersample π (rather than the counts of individual nucleotides [or haplotypes] as above). This approach allows adequate amino acid diversity calculations also when having alleles >1 bp (haplotypes). The gene-wise amino acid

level F_{ST} is calculated analogously to the gene-wise nucleotide level F_{ST} from the amino acid intra- and intersample π values. As for gene-wise nucleotide diversity, a gene will for a sample get π = NA if one or several loci in the gene that are included in the VCF file have missing data for the sample.

1.4. *pN/pS*

pN/pS measures the ratio of the nonsynonymous to the synonymous polymorphism rates, where *pN* equals the fraction of possible nonsynonymous mutations that are observed as polymorphisms and *pS* equals the fraction of synonymous mutations that are observed as polymorphisms. To calculate the *pN/pS* for a gene and sample, POGENOM first derives a consensus nucleotide sequence for the gene in the sample by modifying the reference nucleotide in variant loci based on the most frequent allele, while keeping the reference sequence in invariant positions. For each nucleotide position, every possible (single nucleotide) mutation relative to the consensus sequence is then recorded, and whether this mutation is nonsynonymous or synonymous and present as a polymorphism or not.

pN/*pS* is then calculated as:

$$pN/pS = \frac{\left[\frac{\sum_{i=1}^{L} n_i}{\sum_{i=1}^{L} N_i}\right]}{\left[\frac{\sum_{i=1}^{L} s_i}{\sum_{i=1}^{L} S_i}\right]}$$

where n_i is the number of observed nonsynonymous mutations (alleles), N_i is the total number of possible nonsynonymous mutations, s_i is the number of observed synonymous mutations and S_i is the total number of possible nonsynonymous mutations for locus *i.* If no synonymous mutations are observed for the gene, *pN/pS* is set to NA. In addition to calculating *pN/pS* on a per-sample basis, POGENOM calculates it on all samples collectively by combing the allele frequencies of all samples.

2. Supplementary figures

Supplementary figure 1. Nucleotide diversity (π) over time of five BACLs with at least eight samples in the LMO data set (upper figure). Lower panel compares difference in seasonal time (in days) between samples against difference in π. We define difference in season time as the shortest distance between two dates of the year, i.e. January 2 and December 30 have a difference in seasonal time of 3 (independent of which years they were from).

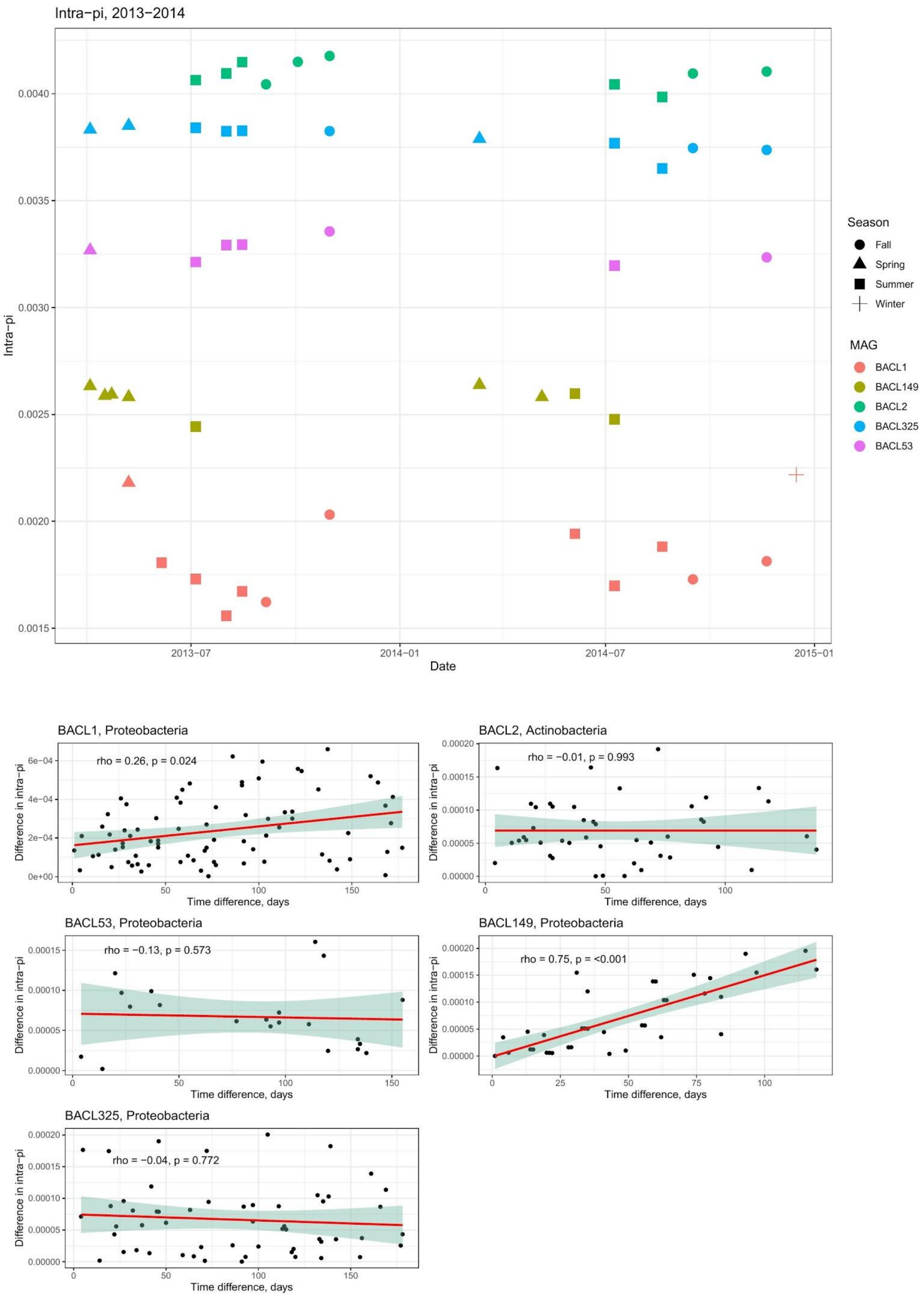

Supplementary figure 2. Comparison of F_{ST} obtained at different sequence depths for one genome. The read-mapping (BAM) files for 21 samples for BACL53 were downsampled to either 80X, 40X, 20X or 10X median coverage depth. Variant calling was run on the downsampled files (after combining them, see Methods) and POGENOM was run with '--min_count' and '--subsample' set to 40, 20, 10 or 2 (i.e. the total allele counts for each locus was downsampled to these levels). The later numbers are what "Depth" refers to in the figure. Each data point is the estimated genome-wide F_{ST} for a pair of samples. "r" is the Pearson correlation coefficient for the comparison. Depth_10 is the level used in this study.

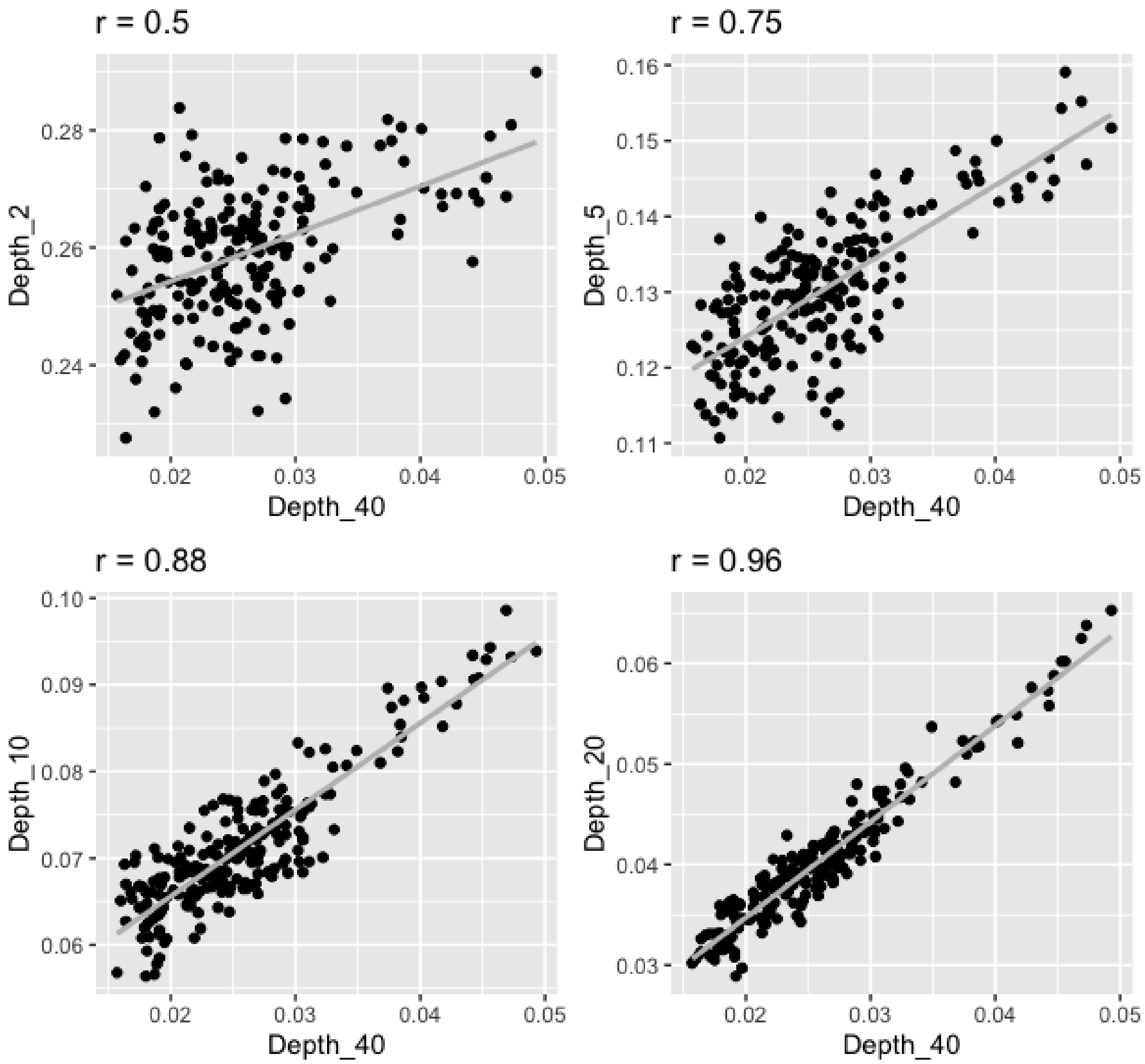

Supplementary figure 3. Mean pN/pS of single-copy core genes (SCG) compared against all other genes in the respective BACL.

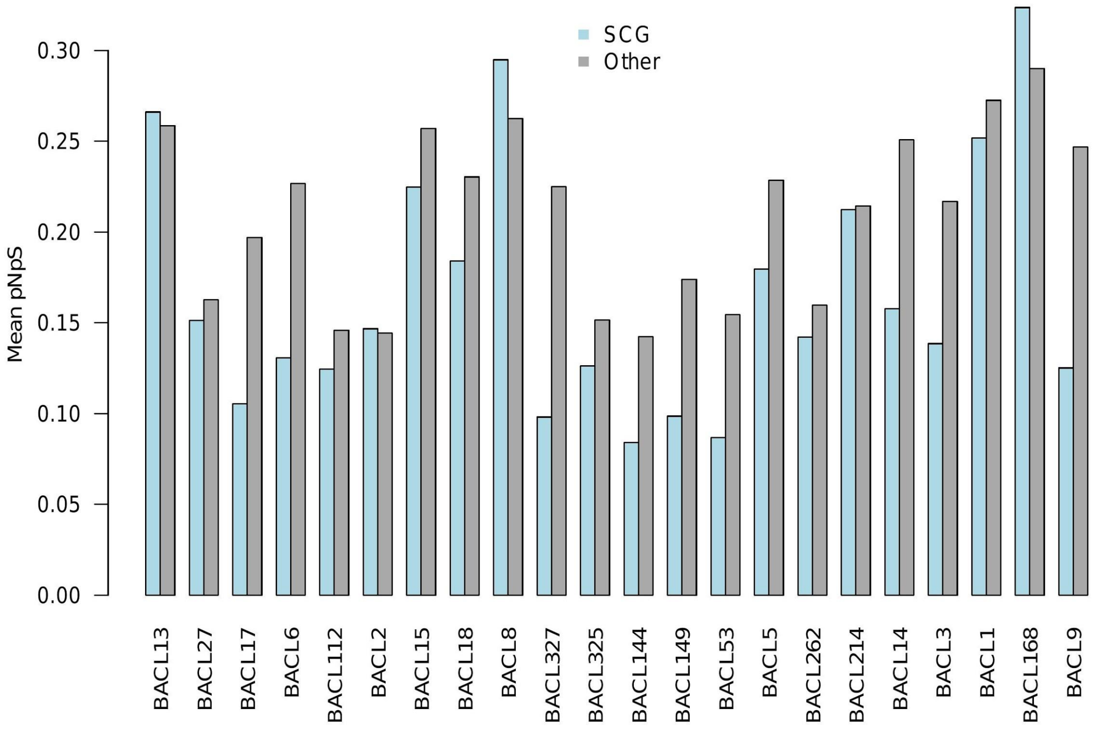

Supplementary figure 4. KEGG pathway map00910. Genes with significant F_{ST} are highlighted in red.

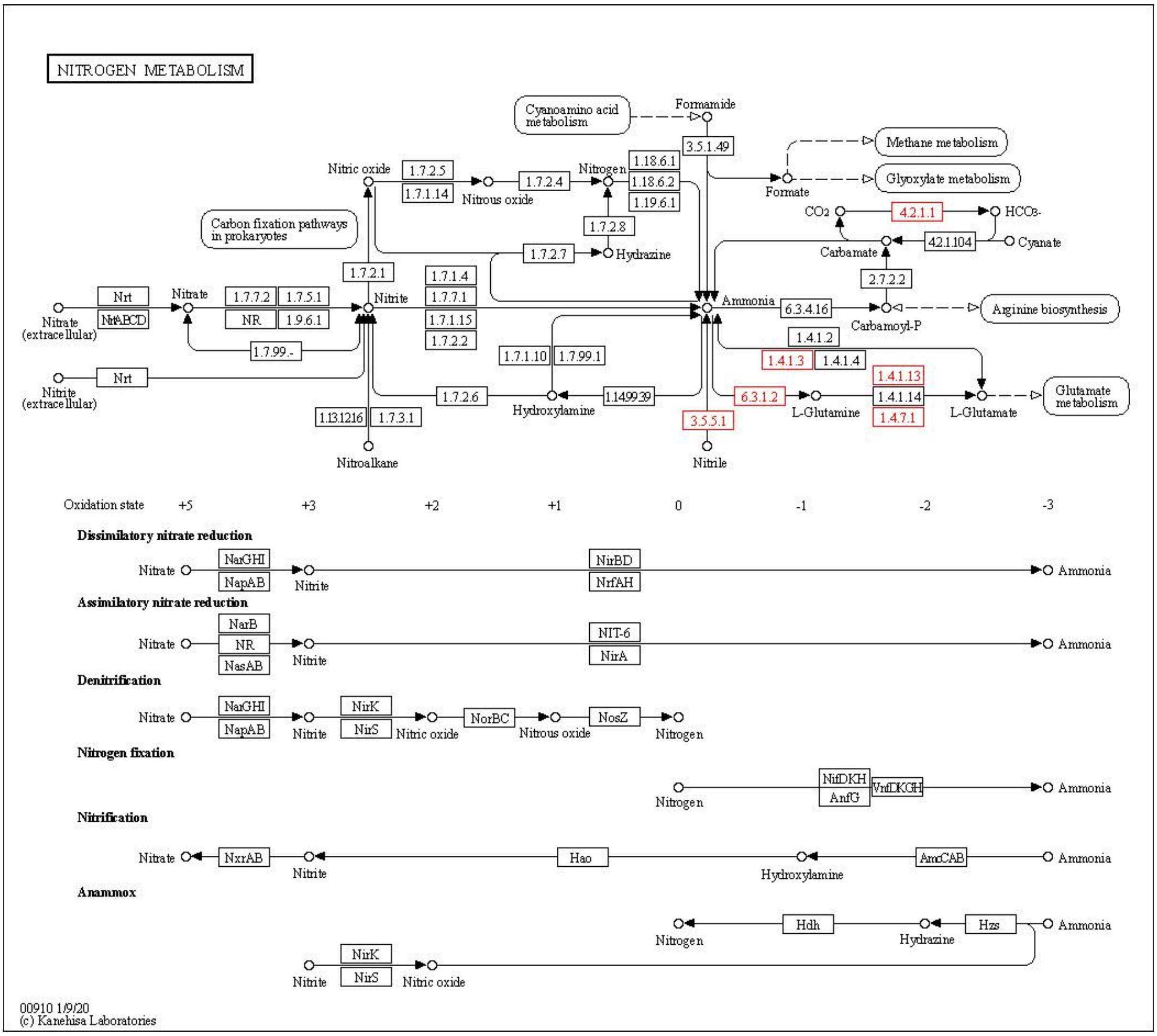

Supplement: Supplementary file 1 — Suppl. figures and info [file 41396_2021_985_MOESM1_ESM.pdf]
